# Expression of estrogen receptors, PELP1, and SRC in human spermatozoa and their associations with semen quality

**DOI:** 10.1007/s13577-022-00847-6

**Published:** 2022-12-28

**Authors:** Izabela Skibińska, Mirosław Andrusiewicz, Magdalena Jendraszak, Aleksandra Żbikowska, Piotr Jędrzejczak, Małgorzata Kotwicka

**Affiliations:** Chair and Department of Cell Biology, Poznan University of Medical Sciences, Poznan, Poland

**Keywords:** Estrogen receptors (ESR1 and ESR2), Proline-, glutamic acid-, and leucine-rich protein 1 (PELP1), Proto-oncogene tyrosine-protein kinase c-Src (SRC), Steroid hormones, Sperm

## Abstract

Sperm cells are target cells for both estrogens and xenoestrogens. Due to the specific structure of spermatozoa, these hormonal compounds may act on sperm in a non-genomic mechanism only. However, the ESR-mediated signaling pathways are still poorly understood. In this study, we obtained 119 samples from male participants of Caucasian descent who donated semen for standard analysis. We analyzed gene expression of estrogen receptors (*ESR1* and *ESR2*) and their coregulators—proline-, glutamic acid-, and leucine-rich protein 1 (*PELP1*), and cellular kinase c-Src (*SRC*). RNA level was established using reverse-transcribed RNA as a template, followed by a polymerase chain reaction. Proteins’ presence was confirmed by western blot and immunocytochemistry techniques. “Normal” values of semen parameters were defined as follows: > 32% sperm with progressive motility, > 4% sperm cells with normal morphology, > 15 × 10^6^ sperm per mL, > 58% live spermatozoa and leukocyte amount < 10^6^ cells per mL, according to WHO 2010 reference. Semen parameters that deviated from these “normal” values were labeled as “abnormal”. Gene expression ratios revealed significant, moderate, and negative correlations for *ESR1*/*ESR2* and weak, negative *ESR2*/*PELP1* correlations in the subgroup of patients with abnormal values of semen parameters. In addition, SRC/PELP1 was moderately and positively correlated in the subgroup with parameters within the reference values established by WHO 2010. Our study showed that both PELP1 scaffolding protein and SRC kinase might influence semen quality via ESRs. It seems that not the expression of a single gene may affect the sperm quality, but more gene-to-gene mutual ratio. Characterization of estrogen-signaling pathway-related genes’ modulated expression in sperm cells could aid in better understanding sperm biology and quality.

**Supplementary Information:**

The online version contains supplementary material available at 10.1007/s13577-022-00847-6.

## Introduction

Reproductive medical care for men has lagged behind that of women. Even though progress has been made in the field of male reproductive health, idiopathic male infertility remains a challenging condition to diagnose and manage. Very often, it manifests with altered semen characteristics without an identifiable cause. Nearly, half of all infertility cases are due to a male factor, contributing to 30% of these cases and another 20% of cases if combined with female factors [1–3]. The interplay of thousands of genes, proteins, and metabolites contributes to male infertility problems. Etiological factors cannot be identified in ~ 70% of the male partners of infertile couples, with the cause of male infertility remaining elusive [1, 2]. Nearly, 10−15% of men in their prime reproductive age are affected by so-called idiopathic male infertility, whose etiology remains unknown. Various genomic studies suggest that more than 500 target genes may be associated with this disease condition [4].

Semen analysis remains a primary diagnostic tool for male infertility [5–7]. Given the high variability in standard semen parameters, identification of other additional useful biomarkers has been explored, including those for DNA damage, anti-sperm antibody testing, as well as gamete interaction tests (acrosome reaction testing, sperm penetration assays, sperm-zona pellucida binding assessments, and hyaluronan binding assays) [1, 8, 9]. However, semen analysis remains an imperfect test. Its results can be influenced by physiologic parameters, including medical comorbidities, abstinence intervals, systemic illness, or lifestyle factors such as work environment, physical activity, diet, and environmental exposures [10–13]. The listed factors can influence most of the results of medical tests performed in assessing the reproductive potential of men and women, not only semen analysis. Therefore, seminological diagnostics should be developed and extended using new biomarkers because the standard semen analysis is insufficient to explain the causes of infertility in many clinical cases.

A review of the published literature over the past few years makes it rather evident that current research concerning male infertility has become more focused on assessing sperm functions at the molecular level. It was proved that estrogens play an important role in spermatogenesis and that spermatozoa are the target cells for estrogens. Not only are sperm cells able to synthesize estrogen, but both estrogen receptors are also expressed in spermatogenic cells at different stages (from spermatogonia to spermatozoa). However, the cellular localization is not identical—both receptor types are located in the midpiece, while ESR2 is also expressed in the tail. In addition, in ejaculated sperm, 17beta-estradiol stimulates functions such as motility, vitality, acrosome reaction, and capacitation. Estrogen-induced biochemical changes occur rapidly, i.e., during capacitation. It suggests the non-genomic action of these hormones in spermatozoa [2, 14, 15].

Maintaining the balance between estrogens and androgens is essential for the proper functioning of the male reproductive system [16]. The observed levels of ESR1/ESR2 expression depend on the male’s tissue type and age [17, 18]. Studies indicate greater male exposure to endogenous and exogenous estrogens in recent years. It may influence male reproductive biology [19–24]. Exogenous estrogens are widely distributed in the environment and can be found in plastics, cosmetics, drugs, detergents, and food [16]. This category also includes endocrine-disrupting chemicals present in the environment. These estrogen-like compounds and anti-androgenic chemicals include e.g., dioxins, some pesticides, polychlorinated biphenyls, and dichlorodiphenyltrichloroethane. They can mimic the action of endogenous estrogens and interfere with any aspect of their function, which is crucial in maintaining homeostasis and regulating developmental processes. This way, they adversely affect the endocrine system and male reproductive health [25, 26]. However, the exact molecular mechanism of action remains vague [27–31]. The concept of “chemical mixture effects” has been widely discussed in this context to indicate that combined chemical action may induce different biological effects than an individual chemical’s influence [32–35].

It was reported that the biological effects of estrogens and xenoestrogens might occur via genomic and rapid, non-genomic pathways, which also involve a wide range of coregulators. Both types of classical estrogen receptors (ESRs; ESR1/ESR2) and G protein-coupled estrogen receptor 1 (GPER, GPR30) have been confirmed in different tissues of the male reproductive tract, spermatogenic cells, and spermatozoa. ESRs expression pattern was studied not only in samples obtained from male patients but also in murine models. ESR2 in the seminiferous tubule is expressed in all spermatogenic cells (i.e., spermatogonia, spermatocyte, round and elongated spermatids) and Sertoli cells. ESR1 expression is very similar, except for spermatids [36, 37]. ESRs are mostly involved in genomic and rapid signaling, while GPER mainly mediates non-genomic effects [28, 31, 38–44]. Apart from these well-known receptors, it has been suggested that other novel proteins may play a similar role in mediating estrogen signaling in males. These proteins are often characterized as putative estrogen receptors and include estrogen-related receptor, saxiphilin-binding protein, ER-X, and ER-x [45–50]. It is important to point out the role of estrogen-signaling coregulators that mediate the estrogen response at the tissue and cellular level, i.e., the novel scaffolding protein, proline-, glutamic acid-, and leucine-rich protein 1 (PELP1), also known as MNAR (modulator of non-genomic action of estrogen receptor). In addition to nuclear receptors and transcription factors (such as activator protein 1, specificity protein 1, or nuclear factor kappaB), PELP1 was proven to interact with several key players of cell cycle progression. These include epidermal growth factor receptor, phosphoinositide 3, and proto-oncogene tyrosine-protein c-Src (SRC) kinases [51–56]. It was reported that activation of SRC and SRC-mediated signaling is coordinated by binding of PELP1 and ESR to SRC’s SH3 and SH2 domains, respectively, and is stabilized by the ER-PELP1 interaction through PELP1’s LXXLL motifs [54]. This interaction potentially provides PELP1’s additional level of steroid hormone regulation of cell and/or tissue functions and may affect sperm cell biology.

Our team has already confirmed PELP1 expression in human sperm cells. We established that the elevated expression might be related to deteriorated sperm parameters [57]. Therefore we decided to investigate further whether there is any relationship between estrogen receptors, PELP1, SRC kinase, and sperm parameters, as it may advance our understanding of sperm biology and potentially male fertility.

## Materials and methods

### Participants

Semen samples were obtained between 2018 and 2020 from 119 participants of Caucasian descent enrolled from the general population with unknown fertility status. They were admitted by the physicians to the Division of Infertility and Reproductive Endocrinology of Poznan University of Medical Sciences (PUMS), due to conception problems, for in-depth andrological consultation. All study participants were of reproductive age. The men who underwent a first-time seminological study and had no knowledge of semen quality (no previous seminological studies) were recruited. Male patients with erectile disorders who could not provide sperm samples during masturbation were excluded from the examinations. To obtain the most homogeneous group, recruited individuals not only complied with the recommended duration of sexual abstinence, from 2 to 7 days but most often, it was a 4-day period. In addition, the men with varicocele, prostate dysfunction, vas deferens obstruction, and metabolic diseases were excluded from further molecular investigations. This study did not analyze the data on having offspring in the participants’ group; therefore, men with reduced semen quality were not necessarily infertile. Patients with fever (≥ 38.5 °C) in the last three months prior to the study were excluded from the study, and if they reported any medical conditions or procedures that might have caused infertility in their history: varicocele, prostate dysfunction, vas deferens obstruction, as well as metabolic diseases, urogenital surgery, scrotal injury, genetic diseases (cystic fibrosis, Kartagener syndrome), cryptorchidism, endocrine hypogonadism, previous radiotherapy or chemotherapy, exposure to diagnostic X-rays, inguinal hernia surgery, suspected urogenital tract infection, chronic parotitis at age 13 and above, chronic diseases or drugs with a gonadotoxic effect, such as psychotropic drugs, antiepileptic drugs or cardiac drugs.

### Methods

#### Semen analysis

Semen analysis was performed manually following “The WHO Laboratory Manual for the Testing and Processing of Human Semen, 5th ed. 2010” [58] under the supervision of an expert in the field of laboratory diagnostics. “Normal” values of semen parameters were referenced as follows: > 32% sperm with progressive motility, > 4% sperm cells with normal morphology, > 15 × 10^6^ sperm per mL, > 58% live spermatozoa, and leukocyte amount < 10^6^ cells per mL. Semen parameters below “normal” WHO reference ranges were defined as “abnormal”.

All analyses were performed using an Axioskop 2 microscope (Zeiss, Carl Zeiss AG, Oberkochen Germany). To determine sperm concentration improved Neubauer hemocytometer chamber (Blaubrand, Brand GmbH, Wertheim, Germany) was used. We focused on the relationship between normal/abnormal parameter values and gene expression; thus, we did not divide patients into groups with normozoospermia, oligozoospermia, asthenozoospermia, etc.

Among 119 participants aged from 23 to 47 years (mean 34 ± 5 years), in 86 patients, at least one semen parameter deviated from the reference range. In 33 participants, all analyzed sperm parameters were within WHO range values [58]. Participants’ clinical characteristics are described in “Results” and Table 1.

**Table 1:** Participants’ clinical characteristics and subdivisions according to normal and abnormal semen parameters

	All participants					Participants with sperm parameters within WHO 2010 reference range (normal)					Participants with at least one sperm parameter outside the WHO 2010 reference range (abnormal)					*p* value
	*N*	Mean	SD	Min	Max	*N*	Mean	SD	Min	Max	*N*	Mean	SD	Min	Max	
Concentration [106 per ml]	119	43.4	29.5	2.5	125	33	63.8	27.3	16	125	86	35.6	26.5	2.5	93	** < 0.0001**^**c**^
Progressive motility [PR, %]	119	38.1	14.0	5	67	33	46.5	10.4	32	66	86	34.8	13.9	5	67	** < 0.0001**^**a**^
Total motility [PR + NP, %]	119	59.6	13.4	15	90	33	65.9	9.3	47	90	86	57.2	14	15	85	**0.0002**^**b**^
Immotile spermatozoa [IM, %]	119	40.4	13.4	10	85	33	34	9.2	10	53	86	42.7	14	15	85	**0.0002**^**b**^
Vitality [%]	119	73.9	11.0	41	95	33	78.5	7.2	62	95	86	72.1	11.7	41	95	**0.0005**^**b**^
Morphology [%]	119	2.6	2.3	0	11	33	5.5	1.9	4	11	86	1.5	1.2	0	5	** < 0.0001**^**b**^
Round cells [106 per ml]	119	0.8	0.5	0	2.5	33	0.9	0.5	0	2.2	86	0.7	0.6	0	2.5	**0.0089**^**c**^
Round cells [%]	119	2.2	2.0	0	11	33	1.6	1.2	0	6.3	86	2.4	2.2	0	11	0.1018c
RNA (ng/μL)	117	323.2	309.7	13.1	1427.5	32	336.0	293.8	13.9	1248.6	85	318.4	317.0	13.1	1427.5	0.2441^c^
*ESR1*^*‡*^	9.40E + 01	2.10E− 02	3.17E− 02	0	1.34E− 01	30	3.0E− 02	3.8E− 02	0	1.3E− 01	64	1.7E− 02	2.8E− 02	0	1.3E− 01	0.0681^c^
*ESR2*^*‡*^	9.20E + 01	2.44E− 03	6.19E− 03	0	3.58E− 02	30	1.9E− 03	6.5E− 03	0	3.6E− 02	62	2.7E− 03	6.1E− 03	4.2E− 06	3.2E− 02	0.1371^c^
*SRC*^*‡*^	9.20E + 01	1.42E− 02	7.32E− 02	0	5.64E− 01	29	1.9E− 02	1.0E− 01	0	5.6E− 01	63	1.2E− 02	5.4E− 02	0	3.1E− 01	0.4493^c^
*PELP1*^*‡*^	9.00E + 01	2.81E− 03	1.41E− 02	0	1.13E− 01	27	4.9E− 04	2.5E− 03	0	1.3E− 02	63	3.8E− 03	1.7E− 02	0	1.1E− 01	0.3536^c^

Statistically significant differences are indicated in bold

*N* number of cases, *SD* standard deviation, *Min and Max* minimal and maximal values, *E* 10 to the power of E (exponent), *PR* progressive motility, *NP* non-progressive motility, *IM* immotile sperm

^a^*t* Student test

^b^*t* Student test with separate variance estimates

^c^Mann–Whitney *U* test

^‡^Gene relative expression level expressed as reference gene’s and standard curve normalized values

#### Nucleic acid isolation and validation

We aimed to obtain total high molecular weight RNA from the whole fraction of sperm cells, irrespective of their parameters. Thus, we did not use standard isolation techniques (e.g., Percoll, swim-up) from the whole fraction of sperm cells. We did not perform multiple centrifugations to avoid RNA degradation [59]. Semen was washed gently with an equal volume of phosphate-buffered saline (Corning Incorporated, Corning, NY, USA) and pelleted by centrifugation (0.6 × g, 5 min, room temperature). The semen samples were incubated on ice for half an hour in the somatic cell lysis buffer (0.1% SDS, 0.5% Triton X-100 in ddH_2_O; LabEmpire; Rzeszów, Poland) to lyse the round cells [60, 61] and centrifuged to obtain sperm cells’ fraction. The absence of round cells was established by light-microscopic evaluation. RNA extraction from sperm was performed using 500 μL of acid-guanidinium-phenol reagent (AGP; GenoPlast Biochemicals; Rokocin, Poland). The samples were incubated with gentle shaking in the AGP (250 rpm, 15 min, 55 °C) to separate the proteins and cellular debris. Subsequently, to allow the DNA, RNA, and protein phase-separation, 200 μL of chloroform (Avantor Performance Materials Poland S.A., Gliwice, Poland) were added to the AGP mixture, incubated (3 min, RT), and centrifuged (12,000 × g, 15 min, RT). The aqueous, upper phase was transferred into a new Eppendorf tube, and an equal volume of cold (− 20 °C) absolute ethanol (Avantor Performance Materials Poland S.A., Gliwice, Poland) was then added and thoroughly mixed by inverting the tube a few times. The mixture was transferred into a Direct-zol RNA Miniprep silica matrix column (ZymoResearch; Irvine, CA, USA), and the HMW-RNA was isolated as recommended by the manufacturer. Quantity, quality, and purity were analyzed in compliance with the previously described methodology [62] using a NanoPhotometer^®^ NP-80 UV/VIS Touch (IMPLEN; Munich, Germany). RNA integrity was evaluated using electrophoretic separation under denaturing conditions. The isolated RNA was immediately stored at − 80 °C until further analysis.

#### Reverse transcription and quantitative PCR

According to the manufacturer’s instructions, a three-step transcriptor reverse transcriptase reaction was performed to synthesize DNA to complementary RNA (cDNA) (Roche; Manheim, Germany). We incubated a mixture of 5 mM oligo(d)T_10_, 1 mM random hexamer primer (Genomed; Warsaw, Poland), 1 μg HMW-RNA, and RNase-, DNase- and pyrogen-free water (Thermo Fisher Scientific; Waltham, MA, USA) for 10 min at 65 °C. Subsequently, samples were chilled on ice. The HMW-RNA mixture was supplemented with 10 U/rx transcriptor reverse transcriptase, 5 U/rx ribonuclease inhibitor, 1 × reaction buffer (Roche; Manheim, Germany), 0.1 U/μL E. coli poly(A) polymerase, 0.1 mM adenosine triphosphate (New England BioLabs; Ipswich, MA, USA), and 100 mM deoxyribonucleotide triphosphates (Novazym; Poznan, Poland). The total volume of the cDNA reaction mixture was 20 μL. The subsequent steps of cDNA synthesis were followed as described previously [62]. All cDNA samples were synthesized in duplicates and immediately used for qPCR reactions or stored in − 20 °C until further analysis but no longer than seven days.

The quantitative PCR reactions were designed in line with the MIQE guidelines [63] (Online Resource 1, MIQE checklist and data set). The Roche Universal ProbeLibrary (UPL) Assay Design Center (http://qpcr.probefinder.com, last accessed on September 28, 2017) was utilized to determine primer sequences and TaqMan^®^ hydrolysis probe positions for the following genes of interest: *ESR1* (F: ccttcttcaagagaagtattcaagg (intron spanning); R: attcccacttcgtagcatttg; probe #69, Roche cat. no.: 04688686001; amplicon length: 160 bp; GenBank sequence accession numbers: NM_001122740.2, NM_001122741.2, NM_001385571.1, NM_001291241.2, NM_001385568.1, NM_001385572.1, NM_001385570.1, NM_001385569.1, NM_000125.4, NM_001328100.2, NM_001291230.2), *PELP1* (F: caaggaggagactcacaggag (intron spanning); R: caaggaggagactcacaggag; probe #24, Roche cat. no.: 04686985001; amplicon length: 131 bp; accession numbers: NM_014389.3, NM_001278241.2), and *SRC* (F: gccatgttcactccggttt; R: cagcgtcctcatctggtttc (intron spanning); probe #21, Roche cat. no.: 04686942001; amplicon length: 100 bp; accession number: NM_005417.5) [64]. The primers were obtained from Genomed (Warsaw, Poland). Regarding *ESR2* and *HPRT1* reference control gene, we applied ready-to-use assays (PrimePCR, qHsaCEP0052206, BioRad; Hercules, CA, USA and UPL102079, Roche, Manheim, Germany, respectively). The *HPRT* was used as the most stable of the three reference genes taken under consideration. To assess and evaluate all RNA expression patterns, the LightCycler^®^ 2.0 carousel glass capillary-based system was used (Roche Diagnostics International AG; Rotkreuz, Switzerland).

As described previously, all quantitative polymerase chain reactions (qPCR) were made in a total volume of 20 μL [62] with standard cycling and acquisition steps. Quantitative PCR analysis was performed using the appropriately adjusted and standardized reaction mixtures for Roche UPL probes [62] and 1 × LightCycler^®^ FastStart TaqMan^®^ Probe Master Mix (Roche; Manheim, Germany) according to the manufacturer’s protocol. All reactions were conducted in duplicates. The following actions were performed to avoid contamination with DNA-derived artifacts: non-reverse-transcribed RNA we used as a template instead of cDNA in negative controls and the commercial sets of intron-spanning probes or primers.

For each gene, reaction efficiencies were derived from the relevant standard curves [62]. These values were then compared with the appropriate threshold values and reference gene assays using LC 5.0.0.38 software. All results were presented as concentration ratios (Cr—fluorescence measurement results normalized to standard curves and compared to the *HPRT* reference gene), corresponding to the relative expression level [65]. Mean values were then evaluated with the use of relevant statistical analyses.

#### Western blot

For ten randomly selected semen samples, sperm cells were incubated in RIPA Lysis Buffer (Merck KGaA, Darmstadt, Germany). Total extracts were shaken gently (30 min at 4 °C) and centrifuged (10,000 × g, 20 min, 4 °C). The protein-containing supernatant was collected, and protein concentration was assessed using QuickStart Bradford 1 × Dye Reagent (BioRad, USA) and NanoPhotometer^®^ NP80 (IMPLEN, Munich, Germany). Subsequently, 20 µg of protein lysate per lane was diluted with Laemmli buffer (BioRad; Hercules, CA, USA), denatured (80 °C, 10 min), and loaded onto an SDS-polyacrylamide gel (TGX FastCast Acrylamide Kit 10%; BioRad; Hercules, CA, USA). After electrophoretic separation (90 min, 120 V), proteins were transferred (90 min., 0.3 A, 4 °C cold buffer, cooling cartridge) to the activated Immobilon PVDF membranes (Merck KGaA, Darmstadt, Germany) in methanol (Avantor Performance Materials, Poznan, Poland). Subsequently, the membranes were incubated with gentle shaking (200 rpm, 1 h, RT) in blocking buffer (TBS-T; Abcam; Cambridge; UK), supplemented with 5% blotting-grade blocker (BioRad; Hercules, CA, USA). An overnight incubation (4 °C, 200 rpm gentle shaking) was used with primary antibodies against: ESR1 (1:1000, LS-C88420; Lifespan Biosciences; Seattle, WA, USA), ESR2 (1:1000, ab3576; Abcam; Cambridge, UK), PELP1/MNAR (1:1000, A300-180A; Bethyl; Montgomery, TX, USA), SRC (1:1000, orb379229; Biorbyt; Cambridge, UK), p-SRC (phospho-Tyr529 SRC; 1:1000, orb14869; Biorbyt; Cambridge, UK) and GAPDH (1:2500, sc-25778; Santa Cruz; Dallas, TX, USA). All antibodies were diluted in TBS-T buffer with 3% blotting-grade blocker (BioRad; Hercules, CA, USA). The membranes were washed with TBS-T buffer (3 × ; 5 min, RT) and incubated with secondary polyclonal horseradish peroxidase-conjugated goat anti-rabbit antibody (1:1000, Agilent Dako, Santa Clara, USA) on an orbital shaker (1 h, RT with gentle shaking). Blots were visualized by enhanced chemiluminescence using Clarity Western ECL (BioRad, Herkules, CA, USA), and results were documented using G:BOX (Syngene, Cambridge, UK). The 3-color prestained protein marker (Blirt, Gdańsk, Poland) was used as the molecular mass standard.

#### Immunocytochemistry

Protein localization was assessed using standard immunocytochemical procedures as described previously [57]. Formalin-fixed cell smears were prepared and boiled in a microwave oven twice (2 × 10 min, 200 W, water bath) in citrate buffer (pH 6.0, 0.1 mM citric acid solution, 0.1 mM sodium citrate solution; Avantor Performance Materials; Poznan, Poland). After each step of microwaving antigen retrieval, slides were chilled (20 min, room temperature) and incubated for 3 min in a 3% hydrogen peroxide solution (Avantor Performance Materials, Poznan, Poland). The next step included placing the slides in TBS-T buffer (containing 3% bovine serum albumin, fraction V; Merck KGaA; Darmstadt, Germany) for 1 h at RT. The same primary antibodies were used as described in the western blot section at a 1:100 dilution for all immunocytochemical reactions. Unbounded antibodies were washed in TBS-T buffer (3 × , 10 min, RT). The antigens were visualized using EnVision + /HRP system and 3,3′-diaminobenzidine chromogen (Agilent Dako, Santa Clara, USA). Controls included detection reactions carried out under identical conditions, except that the primary antibodies were replaced by nonimmune serum. The slides were assessed using light microscopy (Olympus CX41 microscope, Olympus Corporation, Tokyo, Japan).

#### Statistical analyses

Results were evaluated using Statistica^®^ Version 13.5.0 software for Windows (TIBCO Software Inc., Palo Alto, CA, USA). Participants with semen parameters within the WHO 2010 reference range were compared to those with at least one or more parameters outside the range. In addition, subdivisions were created to analyze each semen parameter separately (concentration, progressive motility, vitality, and morphology). All continuous variables were checked for outliers and were winsorized if any were present using the equation (mean ± 2 × standard deviations) [66]. Obtained results were described by the mean ± standard deviation, and median [interquartile range] values. The Shapiro–Wilk test was applied for the normality of continuous variables distribution assessment. The parametric *t* Student (also with separate variance estimates) and Mann–Whitney *U* (1- sided and 2-sided) tests were applied for statistical analyses in two groups. The Spearman rank correlation test was applied to evaluate the strength of the correlation coefficient (*R*). The absolute magnitude of the observed correlation coefficient was interpreted as follows: *R* < 0.1—negligible, *R* < 0.4—weak, *R* < 0.7 moderate, *R* < 0.9 strong, and *R* ≥ 0.9 very strong [67]. The Bonferroni–Hochberg correction was applied in the case of multiple testing. Data were considered statistically significant at *p* < 0.05.

## Results

**Fig. 1:**
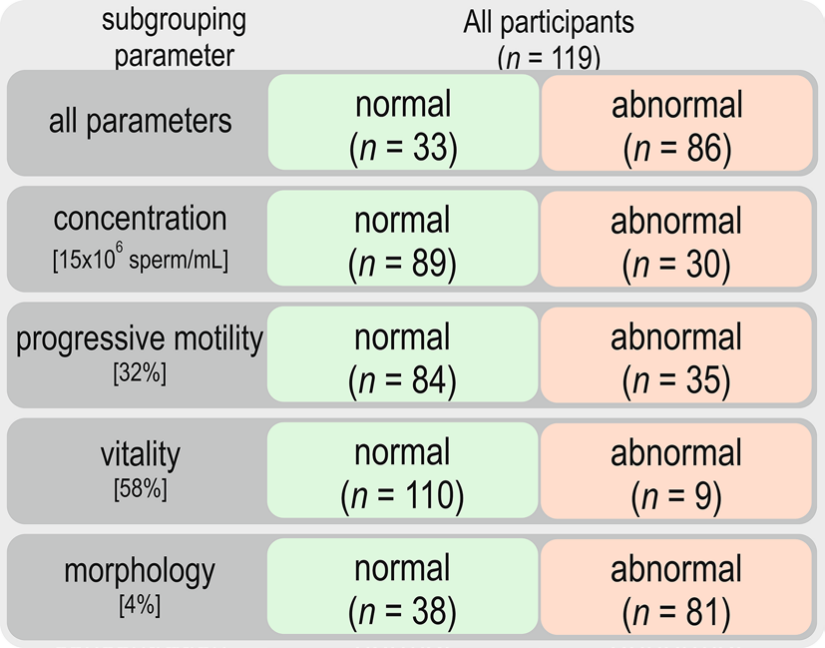
Patient grouping scheme; *n* number of participants. The cutoff points for each parameter are defined in square brackets. Participants above the cutoff points were assigned to the subgroup with a normal value of semen parameter and those below to the abnormal semen parameter value subgroup

The general approach to data inquiry is shown in Fig. 1. If all analyzed semen parameters were within the WHO reference range values [58], men were assigned to the normal basic parameters subgroup; otherwise, they were assigned to the group with at least one or more parameters outside the range (abnormal values of semen parameters subgroup). We wanted to assess whether the level of expression of the given genes is related to changes in sperm parameters. Thus, participants’ subdivisions were created (Fig. 1).

### *ESRs*,* SRC,* and *PELP1* mRNA expression differences

The purpose of the analysis was to establish the expression pattern of genes of interest in relation to sperm parameters. The normalized quantitative mRNA levels were expressed as concentration ratios (Cr values—see “Materials and Methods”).

The patients differed in all analyzed sperm parameters (*p* < 0.05). Amplification of *ESR1, ESR2, PELP1*, and *SRC* was analyzed in all samples. However, genes of interest expression did not differ significantly between normal and abnormal values of semen parameters. The detailed characteristics of semen parameters and mRNA expression levels are provided in Table 1. It is important to note that the differences were observed for particular sperm parameters when these were analyzed separately, as previously mentioned.

Considering the single sperm parameters, apart from vitality, the normalized expression of *ESR1* was higher in subgroups of patients with normal basic parameters, but the differences were insignificant. Regarding *ESR2*, its expression was significantly higher in patients with abnormal values of semen parameters except for vitality. *SRC* was highly expressed in the subgroups of patients with normal standard semen parameters, apart from the motility parameter. In contrast, *PELP1* was highly expressed in the patients with abnormal values of semen parameters, apart from the motility parameter, where its expression was higher when compared to the subgroup of patients with normal standard semen parameters. The differences were not statistically significant (Online Resource 1, Tables S1–S4).

Concerning sperm concentration, the patients differed in analyzed sperm parameters (*p* < 0.05). As expected, the observed values were higher in the subgroup with normal values of semen parameters. The total HMW-RNA concentration obtained from the spermatozoa was significantly higher in patients with normal sperm parameters. The normalized expression of all genes of interest did not differ significantly between subgroups with normal and abnormal values of semen parameters (Online Resource 1, Supplementary Table S1).

With regard to progressive motility, the other sperm parameters differed significantly in all cases (*p* < 0.05) apart from the round cell parameter (*p* = 0.68). Considering the genes of interest expression level, only the expression of *ESR2* differed in patients with abnormal sperm values compared to patients with values within the WHO reference range (*p* = 0.0008). *ESR2* expression was significantly higher in the subgroup with abnormal values of semen parameters (Online Resource 1, Supplementary Table S2).

Regarding vitality, most other sperm parameters differed significantly, apart from morphology and round cell percentage (*p* > 0.05) (Supplementary Table S3). However, there was no significant difference in the expression level of the analyzed genes. Only *ESR2* normalized expression was slightly higher in men with abnormal sperm vitality, but the difference was not statistically significant (*p* = 0.0852; Supplementary Table S3), However, the number of cases was too low to draw far-reaching conclusions.

For morphology, similarly as in the case of the progressive motility subgrouping, the only difference was observed for the round cell percentage, which was lower in the subgroup with normal values of semen parameters (*p* > 0.05; Supplementary Table S4). The normalized expression of *ESR1* and *ESR2* differed between patients with normal and abnormal sperm morphology values (*p* = 0.006, and *p* = 0.0158, respectively; Supplementary Table S4).

### *ESRs*,* SRC,* and *PELP1* gene-to-gene ratios’ expression correlations

**Fig. 2:**
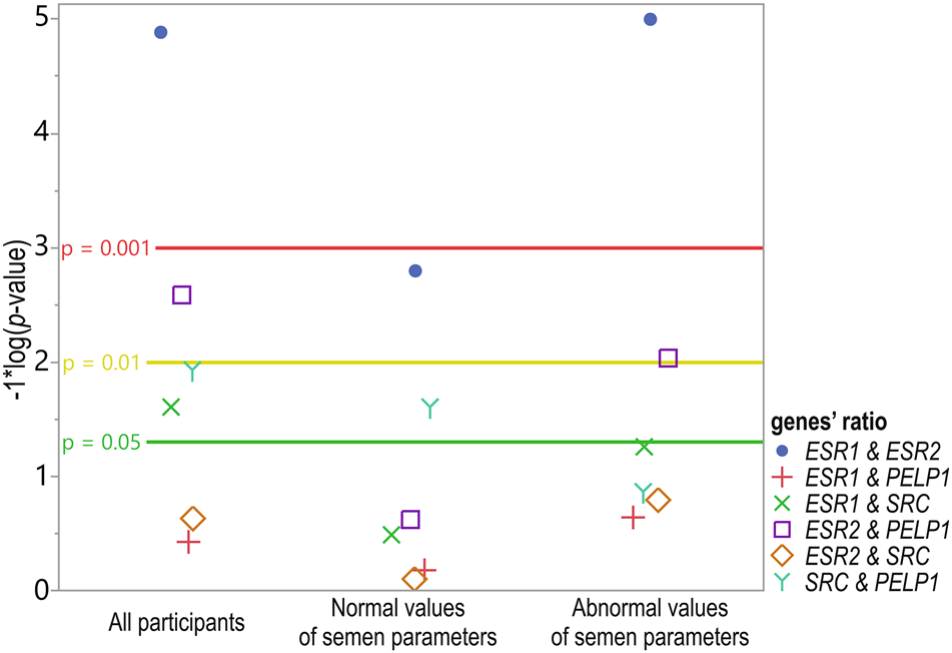
Dot plot showing the correlation coefficients 1*log10 *p* values of all participants (*n* = 109) and in groups with normal (*n* = 33) and abnormal (*n* = 86) values of semen parameters. Reference horizontal lines represent the *p* values thresholds of *p* = 0.05 (green line), *p* = 0.01 (yellow line), and *p* = 0.001 (red line). Participants above the cutoff points were assigned to the subgroup with a normal value of semen parameter and those below to the abnormal semen parameter value subgroup. The cutoff points were as follows: concentration—15 × 10^6^ sperm/mL, progressive motility—32%, vitality—58%, and morphology—4%

When analyzed for all participants, gene-to-gene expression Cr ratios were significantly, negatively, and weakly-to-moderately correlated in most cases, apart from *SRC*&*PELP1*, which was weak and positive. In the case of *ESR1*&*PELP1* and *ESR2*&*SRC*, the correlations were not significant (Fig. 2.; Online Resource 1; Table S5). Considering the Cr ratios in the subgroupings, the gene expression ratios revealed statistically significant, moderate, and negative correlation coefficients for *ESR1*&*ESR2* in both subgroups for all participants (*p* = 0.016; *R* = − 0.55 and *p* < 0.0001; *R* = − 0.55, respectively). Gene expression ratios were also weakly and negatively but significantly correlated in the case of *ESR2*&*PELP1* in the subgroup of patients with at least one abnormal value of semen parameters (*p* = 0.0092; *R* = − 0.33) and moderately and positively correlated for *SRC*&*PELP1* in the subgroup with parameters within the WHO reference range (*p* = 0.0247; *R* = 0.43; Fig. 2.; Online Resource 1 Table S5).

Correlation coefficients for normalized expression were calculated for semen parameters in the subgroups (all parameters within the WHO 2010 reference range vs. at least one or more outside the range) and separately in subdivisions for sperm concentration, progressive motility, vitality, and morphology as previously described.

Correlation coefficients for normalized expression were calculated for semen parameters in the subgroups (all parameters within the WHO 2010 reference range vs. at least one or more outside the range) and separately in subdivisions for concentration, progressive motility, vitality, and morphology as previously described. Regarding *ESR1*, weak associations were observed between expression level and sperm morphology in participants with all parameters within the WHO 2010 reference range vs. those with at least one or more outside the range. The same tendency applied to *ESR1* expression correlation and sperm parameters when analyzed separately for concentration and vitality. The correlation coefficients for the remaining parameters were not significant. Concerning *ESR2*, associations were observed between the gene of interest and progressive motility and morphology parameters in participants with at least one or more parameters outside the range. A weak negative correlation was observed between the gene of interest and progressive motility in patients with normal concentration and vitality values, and a moderate negative correlation was seen in patients with abnormal sperm concentration and vitality values. In addition, a moderate negative correlation was shown for the gene of interest and round cell percentage and morphology in the normal patient subgroup. Other correlations were not statistically significant. For *SRC*, only in patients with abnormal sperm motility moderate positive and negative correlations were observed. Those statistically significant correlations are related to the following parameters: total motility (positive), immotile spermatozoa (negative), and vitality (positive). For *PELP1*, a moderate positive correlation with round cell percentage was found only in patients with abnormal sperm values of motility (Online Resource 1; Supplementary Table S6).

### *ESRs*,* SRC,* and *PELP1* gene-to-gene ratios’ expression correlations with semen parameters

**Fig. 3:**
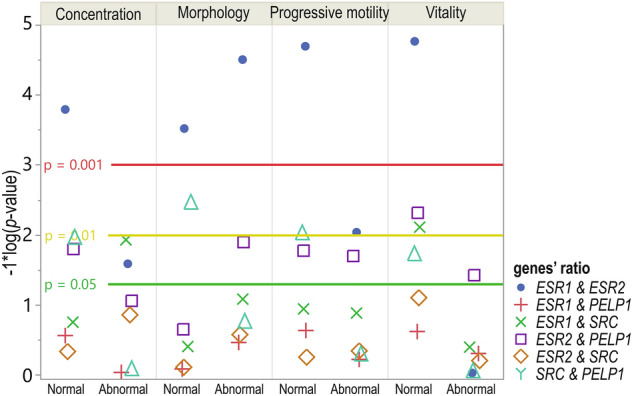
Dot plot showing the correlation coefficients 1*log10 *p* values. Sperm samples were assigned to subgroups with normal or abnormal values of semen parameters: concentration (*n* = 89 vs. *n* = 30), morphology (*n* = 38 vs. *n* = 81), progressive motility (*n* = 84 vs. *n *= 35), or vitality (*n* = 110 vs. *n* = 9). Reference horizontal lines represent the *p* values thresholds of *p* = 0.05 (green line), *p* = 0.01 (yellow line), and *p* = 0.001 (red line)

When analyzed separately for each parameter, gene expression ratios significantly correlated in *ESR1*&*ESR2* regardless of subgrouping, except for vitality, where it was not statistically significant in the group with abnormal values of semen parameters. *ESR1*&*SRC* Cr values were significantly, moderately, and negatively correlated in the subgroup with the abnormal value of the concentration parameter (*p* = 0.0117; *R* = − 0.60) and weakly, negatively correlated in the normal subgroup for the vitality parameter (*p* = 0.0077; *R* = − 0.29). Both *ESR1*&*PELP1*, as well as *ESR2*&*SRC* ratios, showed no significant correlation coefficients. For *ESR2*&*PELP1*, the normalized concentration ratios were negatively and significantly correlated in the case of sperm progressive motility and vitality regardless of subgrouping. For sperm concentration and morphology, correlation coefficients were significant in the subgroup with normal value for concentration parameter (*p* = 0.0158; *R* = − 0.28), and in the abnormal for morphology (*p* = 0.0126; *R* = − 0.9). Interestingly, concerning *SRC*&*PELP1*, the Cr values were significantly correlated for all parameters in the “normal” subgroups (Fig. 3.; Online Resource 1; Table S7).

### ESRs, SRC, and PELP1 protein presence and localization

**Fig. 4:**
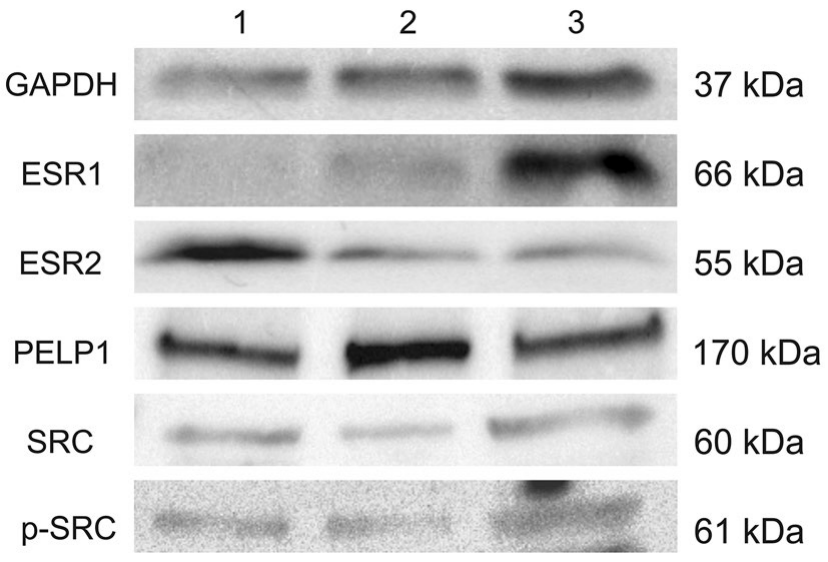
Western blot analysis of analyzed proteins in selected samples. Lines: 1—participant no. 12, 2—participant no. 26, 3—participant no. 70. The proteins’ identities are confirmed by their molecular mass in kilo Daltons (kDa)

The identification of immunoreactive bands confirmed the presence of the analyzed proteins in sperm cells at the expected sizes (ESR1: 66 kDa; ESR2: 55 kDa; PELP1: 170 kDa; SRC: 60 kDa and p-SRC: 61 kDa). The GAPDH reference protein bands (37 kDa) were present in all samples (Fig. 4).

**Fig. 5:**
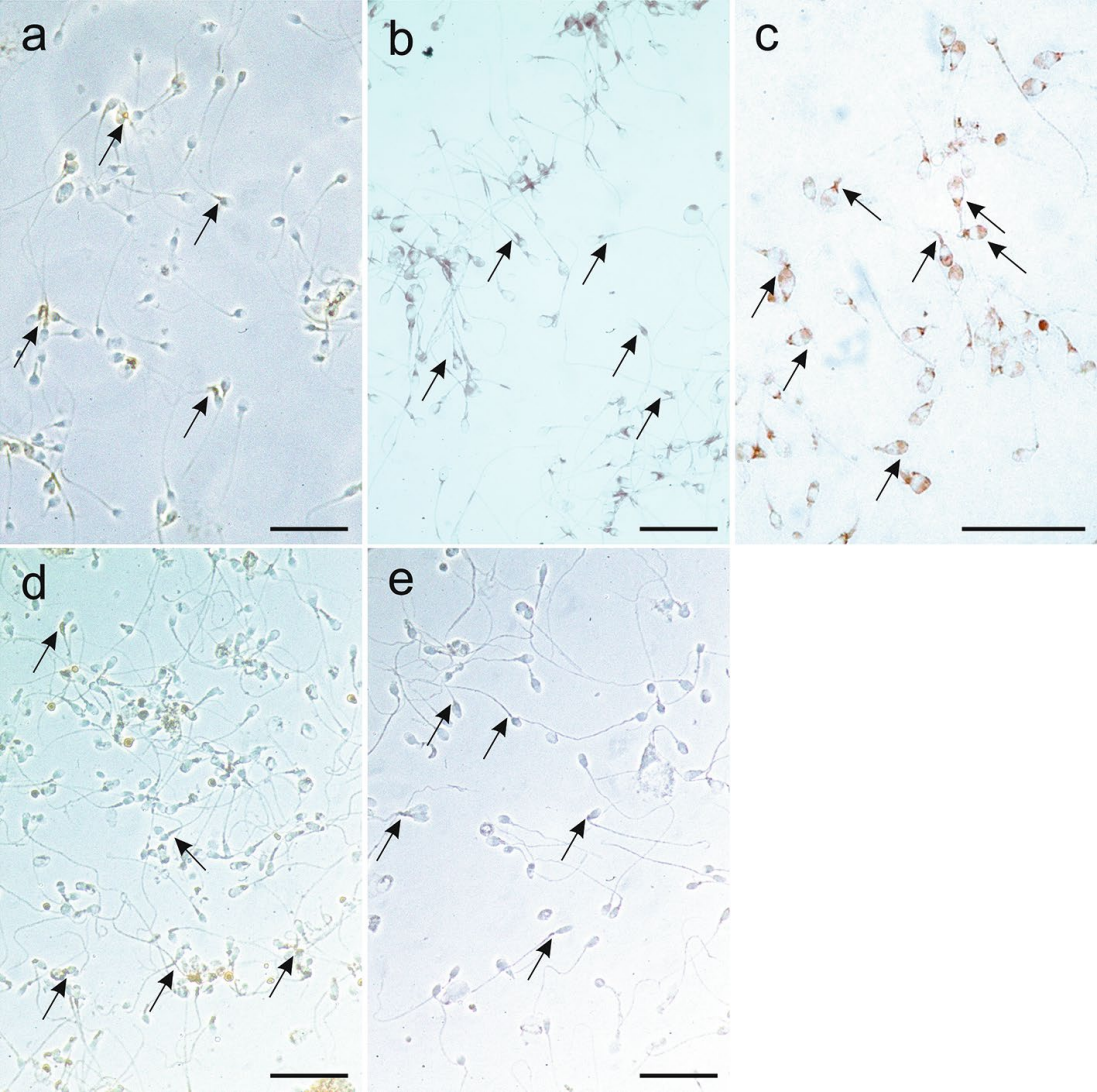
Immunocytochemical staining for analyzed proteins in control samples and analyzed sperm cells. Immunostaining for: **a** ESR1, **b** ESR2, **c** PELP1, **d** SRC, **e** p-SRC. The bar represents 100 μm. The arrows indicate a positive reaction

Immunocytochemical staining enabled the identification of all studied proteins in the cells of interest (Fig. 5). The ESR1 protein was primarily localized in the midpiece and postequatorial part of the spermatozoa head, and ESR2 was identified in midpieces. In comparison, PELP1 was expressed not only in midpieces but also in the acrosome region, including peri-acrosomal space. Similarly, SRC was shown in the midpiece and peri-acrosomal space, while p-SRC was mainly expressed in midpieces.

In the case of all samples, the localization of all described proteins did not differ meaningly between normal and abnormal sperm cells.

## Discussion

In this paper, we confirmed the presence of ESR1, ESR2, PELP1, and SRC transcripts and proteins in the ejaculated spermatozoa. We analyzed potential relationships between the expression of molecules involved in ESR-mediated signaling pathways, such as ESR1, ESR2, and their coregulators, including PELP1 and SRC kinase.

The role of estrogens is indisputable in regulating spermatogenesis and gamete functional maturation. In ejaculated sperm, estrogens may influence sperm functions such as capacitation, acrosome reaction, motility, and vitality. There are also indications that estrogens take part in the spermatogenic regulation of glucose and lipid metabolism [16, 17, 27–29, 42, 68]. As sperm cells are transcriptionally inactive, non-genomic signal transduction via intracellular secondary messengers is the key pathway in these cells. The effects of non-genomic estrogen are mediated via either ESRs or membrane-bound receptors, including GPER, or through interactions with other membrane proteins and/or lipids [27, 69]. PELP1 is considered one of the central modulators of rapid estrogen signaling via estrogen receptors and was reported to interact with several key players of cell cycle progression, i.e., SRC kinases [51–56].

In our study, the expression genes of interest did not differ significantly in participants with all sperm parameters within the reference range compared to those with at least one outside the range. Differences were observed, however, for some parameters when analyzed separately. With regard to ESRs, data evaluating their localization in sperm are not entirely consistent. ESR1 is reportedly expressed in the equatorial and upper post-acrosomal regions of the sperm head and the midpiece. Studies suggest that ESR2 is mainly present in the midpiece region and mitochondria but also in the tail region [70–72]. The mutual localization of ESRs within the midpiece region and in mitochondria implies an essential role of estrogen in sperm motility and metabolism [14, 42]. Our study confirms these findings, as ESR1 protein was primarily localized in the midpiece and postequatorial part of the spermatozoa head, and ESR2 was identified in midpieces. In addition, *ESR1* expression was higher, but not significant, in the participants’ group with the motility parameter values within the WHO range. Only the normalized expression of *ESR2* differed significantly in patients with the abnormal motility parameter value compared to those within the WHO reference range [58]. Interestingly, normalized *ESR2* expression was also higher for most sperm parameters in men assigned to the abnormal subgroup when the expression pattern was analyzed for each parameter separately, except for concentration.

We established that in the group of men with normal sperm morphology parameters, the *ESR1* expression was significantly higher. On the other hand, *ESR2* was also higher in normal subgroups apart from the vitality parameter. It may imply the involvement of estrogen receptors in regulating various cell functions. It may also reflect the spermatozoa polarization in the structure and function, expressed in their ability to sectionalize particular signaling pathways to the target regions. Furthermore, due to the presence of ESRs within the equatorial segment or upper post-acrosomal region, it cannot be excluded that estrogens are involved in the activation of the acrosomal reaction and capacitation in cooperation with SRC. Tyrosine kinase SRC has been identified as a crucial molecule that might mediate rapid estrogen action by physical interaction with ESR1 [73–77]. PELP1 could also be involved in this phenomenon as it was reported to control ESR1-induced SRC activation [51, 54]. Our data seem to confirm such a hypothesis, as we observed a positive correlation between *SRC* and *PELP1* expression ratios in normozoospermic patients. In addition, a positive correlation of these genes was observed in patients with normal sperm morphology, progressive motility, and vitality sperm values. In addition, in patients with altered motility, *SRC* mRNA level was positively associated with sperm progressive motility percentage and negatively with immotile sperm percentage.

Regarding *ESR2*, its normalized expression differed significantly in patients with abnormal motility values compared to those within the WHO reference range [58]. Interestingly, normalized *ESR2* expression was also higher in most subgroups with at least one abnormal value of semen parameters (when the expression pattern was analyzed for each parameter separately), apart from concentration. It seems that estrogen signaling mediated via ESR2 in the presence of PELP1 may contribute to altered sperm characteristics, as we found a significant, weak negative correlation between *ESR2* and *PELP1* in participants with at least one or more parameters outside the range. It was also seen in the normal subgroups of patients for concentration, progressive motility, and vitality parameters. A negative correlation was also observed in patients with altered morphology and motility parameters (weak and moderate, respectively). A strong, negative correlation was observed in patients with abnormal vitality. This observation suggests that altered expression of not one, but the entire ESR-mediated social network signaling pathway, could influence sperm concentration, motility, vitality, and morphology.

*PELP1* expression was higher in participants with at least one or more parameters outside the WHO reference range. This result is consistent with previous investigations aiming to confirm PELP1 presence in human spermatozoa. However, it needs to be mentioned that our previous mRNA analyses showed *PELP1* presence in pooled semen samples. We also observed higher protein expression in samples with abnormal values of semen parameters [57]. In this study, we established a method that allowed us to analyze mRNA expression in samples obtained from single patients.

Our immunocytochemical studies revealed that PELP1 is expressed in the midpieces; it cannot be excluded that its expression may be induced by changed cell altered energy metabolism, i.e., due to oxidative stress. Based on the above results, it may be suggested that the molecular mechanism of the ESR2-PELP1 interaction could be a more susceptible target for endocrine disruptors. However, the literature does not unequivocally confirm the negative impact of xenoestrogens on male fertility. Still, in light of numerous studies, xenoestrogens probably have a significant negative effect on fertility. Studies indicate that exposure to certain concentrations of xenoestrogens correlates with lower sperm quality as well as with testicular carcinogenesis and endocrine disorders, including abnormalities in the level of the reproductive hormones [32, 78–81]. They may increase the number of immature sperm, sperm sex chromosome disomy, and decrease sperm motility. In addition, it has been suggested that estrogen metabolites might increase oxidative stress in human sperm cells [19–21]. On the other hand, papers report that some xenoestrogens may have antioxidant and protective properties concerning sperm cells. Under certain circumstances, some xenoestrogens (phytoestrogens, such as genistein) may influence the ability of sperm to fertilize the egg. It should be mentioned that phytoestrogens have incomparably lower biological activity than endocrine disruptors like bisphenol A, DDT, dioxins, or phthalates [22, 23]. However, it seems that the effects of xenoestrogens on sperm biology predominantly depend on substrate type and concentration [24]. All mentioned examples could support our results concerning significant correlations of *ESR2* with the percentage of progressive motility and morphology in participants with at least one semen parameter outside the WHO reference range, men with normal/abnormal concentration values, and the normal subgroup of participants when vitality is analyzed.

All the above considerations stay in line with the concept of an exclusive modality of estrogen action in spermatozoa. It can be assumed that estrogen sensitivity depends on the availability of ESRs and their coregulators. Several groups have tried to detect ESRs in sperm to clarify the role of estrogens on spermatozoa. Unfortunately, the results are not always consistent. It may be because several splice variants of the described *ESRs*, particularly *ESR2*, are either insufficiently characterized or detected using various methodologies or/and antibodies of different origins. Therefore, it cannot be excluded that an uncharacterized protein sharing homology with ESR2 exists and may cross-react with ESR2 antibodies [27]. Of important note, apart from the well-known classical ESRs and GPER, other novel membrane-associated proteins have been considered estrogen receptors, including estrogen-related receptors, saxiphilin-binding protein, ER-X, ER-x, and putative membrane estrogen receptors. Among many ER-X’s features, those worth mentioning in the context of this discussion are as follows: it has a high affinity for binding estradiol; it has sequence homology to ESR1; it can activate extracellular signal-regulated kinases ERK1/2. ER-x is characterized as a membrane receptor with the ability to mediate estrogen responses in some breast cancer cell lines and, therefore, can influence apoptosis, transcriptional regulation, and growth factor signaling. However, as a consensus has not been reached concerning the definition of an ESR, it is still debatable whether these proteins could be considered estrogen receptors [45–50].

Although the key role of estrogens in the male reproductive system has been substantiated, findings regarding estrogens’ impact on sperm biology are inconclusive. It seems that ESR1, ESR2, PELP1, and SRC may be involved in several molecular pathways concerning estrogen signaling in sperm cells by controlling different activities throughout the spermatozoa lifecycle, positively and negatively influencing their biology. However, unveiling all aspects of their interactions and associations with sperm biology requires further assessment. Since male infertility concerns the interplay of thousands of genes, proteins, and metabolites, it is crucial to investigate other molecular targets that could serve as new biomarkers [1–3]. Establishing the dependencies within estrogen signaling in sperm cells could help answer several questions concerning idiopathic infertility.

In this paper, we confirmed the presence of ESR1, ESR2, PELP1, and SRC transcripts and proteins in the ejaculated spermatozoa. We analyzed potential mRNA and protein expression relations of molecules involved in ESR-mediated signaling pathways, such as ESR1, ESR2, and their coregulators, including PELP1 and SRC kinase.

Our findings suggest that ESR1, ESR2, PELP1, and SRC may be involved in several molecular pathways concerning estrogen signaling in sperm cells, which can control various activities throughout the spermatozoa life cycle. To a small extent, expression of SRC and PELP1 was linked with the sperm parameters in comparison to ESRs differed levels in subgroups of participants with normal and abnormal values of particular semen parameters. It seems that not the expression of a single gene may affect the sperm quality but more gene-to-gene mutual ratio. The phenomenon of genes relation could be linked with their mutual interactions or other molecules that play important roles in the estrogen-signaling pathways. Characterization of estrogen-signaling pathway-related genes’ modulated expression in sperm cells could aid in better understanding sperm biology and quality. Further colocalization studies would need to be performed to establish the particular relationships between these proteins.

## Supplementary Information

Below is the link to the electronic supplementary material.

Supplementary file1 (DOCX 129 KB)

## Data Availability

The datasets used and analyzed during the current study are available from the corresponding author on reasonable request.
