# Supplementary material for: Expression of estrogen receptors, PELP1, and SRC in human spermatozoa and their associations with semen quality
Source: Hum Cell. 2022 Dec 28;36(2):554–67. doi: 10.1007/s13577-022-00847-6 (PMC9947025; doi:10.1007/s13577-022-00847-6)
Supplement: Supplementary file 1 — Supplementary file1 (DOCX 129 KB) [file 13577_2022_847_MOESM1_ESM.docx]

**Expression of estrogen receptors, PELP1, and SRC in human spermatozoa and their associations with semen quality**

**Izabela Skibińska**^*^(ORCID: 0000-0002-7684-028X), **Mirosław Andrusiewicz** (ORCID: 0000-0002-8781-3447), **Magdalena Jendraszak** (ORCID: 0000-0001-8139-9532), **Aleksandra Żbikowska** (ORCID: 0000-0002-6891-8569), **Piotr Jędrzejczak** (ORCID: 0000-0003-2437-7737), **Małgorzata Kotwicka** (ORCID: 0000-0002-9802-374X)

**Supplementary Table S1.** Measures of central tendency and dispersion in participants assigned to the groups regarding concentration parameter – WHO 2010 reference [15x10^6^ per mL].

|  | Normal | | | | | Abnormal | | | | | ***P*-value** |
| --- | --- | --- | --- | --- | --- | --- | --- | --- | --- | --- | --- |
|  | **N** | **Mean** | **SD** | **Min** | **Max** | **N** | **Mean** | **SD** | **Min** | **Max** |  |
| Concentration [10^6^ per ml] | 89 | 54.6 | 25.7 | 16 | 125 | 30 | 10.2 | 3.2 | 3 | 14 | **<.0001^c^** |
| Progressive motility [PR, %] | 89 | 41.1 | 12.9 | 5 | 67 | 30 | 29.3 | 13.5 | 6 | 58 | **0.0001^b^** |
| Total motility [PR+NP %] | 89 | 62.1 | 12.9 | 15 | 90 | 30 | 52.2 | 12.3 | 23 | 71 | **0.0002^c^** |
| Immotile spermatozoa [IM, %] | 89 | 37.8 | 12.9 | 10 | 85 | 30 | 47.8 | 12.3 | 29 | 77 | **0.0001^c^** |
| Vitality [%] | 89 | 76.6 | 9.9 | 43 | 95 | 30 | 65.8 | 10.3 | 41 | 86 | **<.0001^c^** |
| Morphology [%] | 89 | 3.1 | 2.4 | 0 | 11 | 30 | 1.2 | 1.4 | 0 | 5 | **<.0001^c^** |
| Round cells [10^6^ per ml] | 89 | 0.9 | 0.6 | 0 | 2.5 | 30 | 0.4 | 0.4 | 0 | 1 | **<.0001^c^** |
| Round cells [%] | 89 | 1.7 | 1.1 | 0 | 6.3 | 30 | 3.7 | 3.0 | 0 | 11 | **<.0009^c^** |
| RNA (ng/μL) | 88 | 360.6 | 316.2 | 13.9 | 1427.5 | 29 | 209.7 | 262.4 | 13.1 | 1261.7 | **<.0009^c^** |
| *ESR1^‡^* | 77 | 2.1E-02 | 3.3E-02 | 0.0E+00 | 1.3E-01 | 17 | 1.9E-02 | 2.8E-02 | 0.0E+00 | 8.9E-02 | .9922^d^ |
| *ESR2^‡^* | 75 | 2.5E-03 | 6.6E-03 | 0.0E+00 | 3.6E-02 | 17 | 2.3E-03 | 3.8E-03 | 4.2E-06 | 1.1E-02 | .9921^d^ |
| *SRC^‡^* | 74 | 1.5E-02 | 7.9E-02 | 0.0E+00 | 5.6E-01 | 18 | 1.1E-02 | 4.4E-02 | 1.1E-11 | 1.9E-01 | .3304^d^ |
| *PELP1^‡^* | 73 | 2.0E-03 | 1.3E-02 | 0.0E+00 | 1.1E-01 | 17 | 6.2E-03 | 1.7E-02 | 0.0E+00 | 6.7E-02 | .6530^d^ |

^a^ – t-Student test; ^b^ – t-Student test with separate variance estimates; ^c^ – Mann-Whitney U test; ^d^ – 2-sided Mann-Whitney U test; *^‡^ –* relative gene expression level expressed as reference gene’s and standard curve normalized values; E – 10 to the power of E (exponent); *p*-values < 0.05 are indicated in bold.

**Supplementary Table S2.** Measures of central tendency and dispersion in participants assigned to the groups regarding progressive motility parameter – WHO 2010 reference [32%].

|  | Normal | | | | | Abnormal | | | | | ***P*-value** |
| --- | --- | --- | --- | --- | --- | --- | --- | --- | --- | --- | --- |
|  | **N** | **Mean** | **SD** | **Min** | **Max** | **N** | **Mean** | **SD** | **Min** | **Max** |  |
| Concentration [10^6^ per ml] | 84 | 51.4 | 29.1 | 5 | 125 | 35 | 24.2 | 20.6 | 3 | 87 | **<.0001^c^** |
| Progressive motility [PR, %] | 84 | 45.2 | 9.4 | 32 | 67 | 35 | 21.1 | 6.7 | 5 | 30 | **<.0001^c^** |
| Total motility [PR+NP %] | 84 | 65.4 | 8.8 | 47 | 90 | 35 | 45.7 | 12.5 | 15 | 64 | **<.0001^b^** |
| Immotile spermatozoa [IM, %] | 84 | 34.5 | 8.7 | 10 | 53 | 35 | 54.3 | 12.5 | 36 | 85 | **<.0001^b^** |
| Vitality [%] | 84 | 77.6 | 8.7 | 58 | 95 | 35 | 64.9 | 11.0 | 41 | 88 | **<.0001^a^** |
| Morphology [%] | 84 | 3.1 | 2.5 | 0 | 11 | 35 | 1.4 | 1.3 | 0 | 5 | **.0002^C^** |
| Round cells [10^6^ per ml] | 84 | 0.9 | 0.6 | 0 | 2.5 | 35 | 0.5 | 0.4 | 0 | 2 | **<.0001^c^** |
| Round cells [%] | 84 | 2.2 | 2.1 | 0 | 11.0 | 35 | 2.2 | 1.8 | 0 | 8 | .6809^c^ |
| RNA (ng/μL) | 82 | 321.4 | 292.7 | 13.9 | 1427.5 | 35 | 327.5 | 350.8 | 13.1 | 1337.6 | .3334^c^ |
| *ESR1^‡^* | 71 | 2.2E-02 | 3.1E-02 | 0.0E+00 | 1.3E-01 | 23 | 1.9E-02 | 3.4E-02 | 0.0E+00 | 1.3E-01 | .2368^c^ |
| *ESR2^‡^* | 70 | 1.6E-03 | 4.9E-03 | 0.0E+00 | 3.6E-02 | 22 | 5.1E-03 | 8.8E-03 | 2.0E-05 | 3.2E-02 | **.0008^c^** |
| *SRC^‡^* | 70 | 1.4E-02 | 7.6E-02 | 0.0E+00 | 5.6E-01 | 22 | 1.4E-02 | 6.6E-02 | 0.0E+00 | 3.1E-01 | .2988^c^ |
| *PELP1^‡^* | 68 | 3.2E-03 | 1.6E-02 | 0.0E+00 | 1.1E-01 | 22 | 1.6E-03 | 5.1E-03 | 0.0E+00 | 1.8E-02 | .8991^c^ |

^a^ – t-Student test; ^b^ – t-Student test with separate variance estimates; ^c^ – Mann-Whitney U test; ^d^ – 2-sided Mann-Whitney U test; *^‡^ –* relative gene expression level expressed as reference gene’s and standard curve normalized values; E – 10 to the power of E (exponent); *p*-values < 0.05 are indicated in bold.

**Supplementary Table S3.** Measures of central tendency and dispersion in participants assigned to the groups regarding vitality parameter – WHO 2010 reference [58%].

|  | Normal | | | | | Abnormal | | | | | ***P*-value** |
| --- | --- | --- | --- | --- | --- | --- | --- | --- | --- | --- | --- |
|  | **N** | **Mean** | **SD** | **Min** | **Max** | **N** | **Mean** | **SD** | **Min** | **Max** |  |
| Concentration [10^6^ per ml] | 110 | 45.6 | 29.6 | 3 | 125 | 9 | 16.5 | 8.1 | 4 | 28 | **<.0001^d^** |
| Progressive motility [PR, %] | 110 | 39.5 | 13.5 | 5 | 67 | 9 | 20.7 | 6.9 | 10 | 30 | **.0032^a^** |
| Total motility [PR+NP %] | 110 | 61.3 | 12.3 | 15 | 90 | 9 | 39.4 | 9.6 | 26 | 51 | **<.0001^a^** |
| Immotile spermatozoa [IM, %] | 110 | 38.7 | 12.3 | 10 | 85 | 9 | 60.6 | 9.6 | 49 | 74 | **<.0001^d^** |
| Vitality [%] | 110 | 75.8 | 9.0 | 58 | 95 | 9 | 50.6 | 5.2 | 41 | 56 | **<.0001^a^** |
| Morphology [%] | 110 | 2.7 | 2.4 | 0 | 11 | 9 | 1.2 | 0.8 | 0 | 3 | .0630^d^ |
| Round cells [10^6^ per ml] | 110 | 0.8 | 0.6 | 0 | 2.5 | 9 | 0.4 | 0.3 | 0 | 1 | **.0193^d^** |
| Round cells [%] | 110 | 2.2 | 2.0 | 0 | 11.0 | 9 | 2.5 | 1.8 | 0 | 7 | .3036^d^ |
| RNA (ng/μL) | 108 | 319.3 | 302.7 | 13.9 | 1427.5 | 9 | 369.9 | 403.4 | 13.1 | 1261.7 | .9399^d^ |
| *ESR1^‡^* | 88 | 2.1E-02 | 3.1E-02 | 0.0E+00 | 1.3E-01 | 6 | 2.8E-02 | 3.9E-02 | 0.0E+00 | 8.9E-02 | .8505^d^ |
| *ESR2^‡^* | 87 | 2.2E-03 | 5.5E-03 | 0.0E+00 | 3.6E-02 | 5 | 7.4E-03 | 1.4E-02 | 1.7E-04 | 3.2E-02 | .0852^d^ |
| *SRC^‡^* | 86 | 1.5E-02 | 7.6E-02 | 0.0E+00 | 5.6E-01 | 6 | 1.1E-03 | 2.7E-03 | 0.0E+00 | 7.0E-03 | .1423^d^ |
| *PELP1^‡^* | 85 | 3.0E-03 | 1.5E-02 | 0.0E+00 | 1.1E-01 | 5 | 0.0E+00 | 0.0E+00 | 0.0E+00 | 0.0E+00 | .8376^d^ |

^a^ – t-Student test; ^b^ – t-Student test with separate variance estimates; ^c^ – Mann-Whitney U test; ^d^ – 2-sided Mann-Whitney U test; *^‡^ –* relative gene expression level expressed as reference gene’s and standard curve normalized values; E – 10 to the power of E (exponent); *p*-values < 0.05 are indicated in bold.

**Supplementary Table S4.** Measures of central tendency and dispersion in participants assigned to the groups regarding morphology – WHO 2010 reference [4%].

|  | **Normal** | | | | | Abnormal | | | | | ***P*-value** |
| --- | --- | --- | --- | --- | --- | --- | --- | --- | --- | --- | --- |
|  | **N** | **Mean** | **SD** | **Min** | **Max** | **N** | **Mean** | **SD** | **Min** | **Max** |  |
| Concentration [10^6^ per ml] | 38 | 59.2 | 28.6 | 11 | 125 | 81 | 36.0 | 27.1 | 3 | 93 | **<.0001^c^** |
| Progressive motility [PR, %] | 38 | 44.4 | 11.4 | 22 | 66 | 81 | 35.1 | 14.2 | 5 | 67 | **.0006^a^** |
| Total motility [PR+NP %] | 38 | 63.7 | 10.5 | 41 | 90 | 81 | 57.7 | 14.2 | 15 | 85 | **.0337^c^** |
| Immotile spermatozoa [IM, %] | 38 | 36.2 | 10.5 | 10 | 59 | 81 | 42.3 | 14.2 | 15 | 85 | **.0297^c^** |
| Vitality [%] | 38 | 77.5 | 7.6 | 62 | 95 | 81 | 72.2 | 12.0 | 41 | 95 | **.0041^b^** |
| Morphology [%] | 38 | 5.4 | 1.8 | 4 | 11 | 81 | 1.3 | 1.0 | 0 | 3 | **<.0001^c^** |
| Round cells [10^6^ per ml] | 38 | 0.9 | 0.5 | 0 | 2.2 | 81 | 0.7 | 0.6 | 0 | 3 | **.0099^c^** |
| Round cells [%] | 38 | 1.9 | 1.6 | 0 | 9.0 | 81 | 2.3 | 2.1 | 0 | 11 | .2943^c^ |
| RNA (ng/μL) | 37 | 331.7 | 288.0 | 13.9 | 1248.6 | 80 | 319.3 | 320.9 | 13.1 | 1427.5 | .3064^c^ |
| *ESR1^‡^* | 35 | 3.3E-02 | 4.0E-02 | 0.0E+00 | 1.3E-01 | 59 | 1.4E-02 | 2.3E-02 | 0.0E+00 | 9.8E-02 | .**0063^c^** |
| *ESR2^‡^* | 35 | 1.6E-03 | 6.1E-03 | 0.0E+00 | 3.6E-02 | 57 | 2.9E-03 | 6.3E-03 | 4.2E-06 | 3.2E-02 | .**0158^c^** |
| *SRC^‡^* | 34 | 1.7E-02 | 9.7E-02 | 0.0E+00 | 5.6E-01 | 58 | 1.3E-02 | 5.6E-02 | 0.0E+00 | 3.1E-01 | .2041^c^ |
| *PELP1^‡^* | 32 | 9.8E-04 | 3.9E-03 | 0.0E+00 | 1.8E-02 | 58 | 3.8E-03 | 1.7E-02 | 0.0E+00 | 1.1E-01 | .2825^c^ |

^a^ – t-Student test; ^b^ – t-Student test with separate variance estimates; ^c^ – Mann-Whitney U test; ^d^ – 2-sided Mann-Whitney U test; *^‡^ –* relative gene expression level expressed as reference gene’s and standard curve normalized values; E – 10 to the power of E (exponent); *p*-values < 0.05 are indicated in bold.

**Supplementary Table S5.** Spearman rank order correlations of the analyzed gene ratios in participants assigned to the subgroups with normal and abnormal values of semen parameters according to WHO 2010 reference values.

|  | All participants | | | **Participants with all sperm parameters within WHO 2010 reference range  (normal)** | | | **Participants with at least one sperm parameter outside the WHO 2010 reference range  (abnormal)** | | |
| --- | --- | --- | --- | --- | --- | --- | --- | --- | --- |
|  | N | R | *P*-value | N | R | *P*-value | N | R | *P*-value |
| *ESR1* & *ESR2* | 92 | -.55 | <.**0001** | 30 | -.55 | .**0016** | 62 | -.55 | <.**0001** |
| *ESR1* & *SRC* | 91 | -.24 | .**0247** | 29 | -.19 | .3252 | 62 | -.24 | .0551 |
| *ESR1* & *PELP1* | 90 | .09 | .3754 | 27 | -.09 | .6645 | 63 | .15 | .2289 |
| *ESR2* & *SRC* | 89 | .13 | .2338 | 29 | -.05 | .7938 | 60 | .18 | .1611 |
| *ESR2* & *PELP1* | 89 | -.32 | .**0026** | 27 | -.23 | .2389 | 62 | -.33 | .**0092** |
| *SRC* & *PELP1* | 88 | .27 | .**0117** | 27 | .43 | .**0247** | 61 | .19 | .1364 |

N – number of cases; R – Spearman’s rank correlation coefficient; *p*-values < 0.05 are indicated in bold.

**Supplementary Table S6.** Spearman rank order correlations of the analyzed genes in subgroups distinguished according to WHO 2010 value of particular semen parameters analyzed separately.

|  | All participants | | | WHO reference range semen parameters | | | | | | concentration - WHO reference [15x106 per ml] | | | | | | progressive motility - WHO reference [32%] | | | | | | vitality - WHO reference [4%] | | | | | | morphology - WHO reference [4%] | | | | | |
| --- | --- | --- | --- | --- | --- | --- | --- | --- | --- | --- | --- | --- | --- | --- | --- | --- | --- | --- | --- | --- | --- | --- | --- | --- | --- | --- | --- | --- | --- | --- | --- | --- | --- |
|  |  |  |  | Normal | | | Abnormal | | | Normal | | | Abnormal | | | Normal | | | Abnormal | | | Normal | | | Abnormal | | | Normal | | | Abnormal | | |
|  | N | R | p | N | R | p | N | R | p | N | R | p | N | R | p | N | R | p | N | R | p | N | R | p | N | R | p | N | R | p | N | R | p |
|  | ***ESR1*** | | | | | | | | | | | | | | | | | | | | | | | | | | | | | | | | |
| Concentration [10^6^ per ml] | 94 | -.02 | .8120 | 30 | -.11 | .5727 | 64 | -.11 | .3789 | 77 | -.04 | .7122 | 17 | .07 | .7797 | 71 | -.06 | .5944 | 23 | -.12 | .6002 | 88 | -.02 | .8552 | 6 | -.70 | .1248 | 35 | -.20 | .2523 | 59 | -.10 | .4289 |
| Progressive motility [PR, %] | 94 | .10 | .3239 | 30 | .04 | .8270 | 64 | .01 | .9569 | 77 | .14 | .2398 | 17 | -.01 | .9682 | 71 | .02 | .8714 | 23 | -.08 | .7137 | 88 | .12 | .2667 | 6 | -.64 | .1731 | 35 | -.07 | .6767 | 59 | .08 | .5698 |
| Total motility [PR+NP %] | 94 | -.06 | .5770 | 30 | .01 | .9561 | 64 | -.18 | .1591 | 77 | .00 | .9781 | 17 | -.41 | .1024 | 71 | -.13 | .2775 | 23 | -.26 | .2284 | 88 | -.08 | .4726 | 6 | .06 | .9131 | 35 | -.09 | .6142 | 59 | -.10 | .4357 |
| Immotile spermatozoa [IM, %] | 94 | .06 | .5644 | 30 | .01 | .9528 | 64 | .18 | .1591 | 77 | .00 | .9975 | 17 | .41 | .1024 | 71 | .14 | .2596 | 23 | .26 | .2284 | 88 | .08 | .4591 | 6 | -.06 | .9131 | 35 | .10 | .5508 | 59 | .10 | .4357 |
| Vitality [%] | 94 | -.04 | .7031 | 30 | .15 | .4238 | 64 | -.14 | .2554 | 77 | -.02 | .8490 | 17 | -.30 | .2405 | 71 | -.14 | .2430 | 23 | -.04 | .8628 | 88 | -.06 | .5772 | 6 | .04 | .9329 | 35 | .07 | .6877 | 59 | -.13 | .3282 |
| Morphology [%] | 94 | .37 | **.0003** | 30 | .38 | **.0372** | 64 | .35 | **.0043** | 77 | .36 | **.0013** | 17 | .51 | **.0365** | 71 | .35 | .0028 | 23 | .28 | .1936 | 88 | .39 | **.0002** | 6 | .34 | .5057 | 35 | .26 | .1290 | 59 | .25 | .0548 |
| Round cells [10^6^ per ml] | 94 | -.02 | .8791 | 30 | -.09 | .6524 | 64 | -.09 | .4632 | 77 | -.01 | .9087 | 17 | -.07 | .7874 | 71 | -.08 | .5092 | 23 | -.08 | .7162 | 88 | -.03 | .7774 | 6 | -.04 | .9329 | 35 | -.10 | .5712 | 59 | -.12 | .3825 |
| Round cells [%] | 94 | .10 | .3406 | 30 | .09 | .6275 | 64 | .10 | .4109 | 77 | .09 | .4482 | 17 | .02 | .9495 | 71 | .06 | .6014 | 23 | .18 | .4120 | 88 | .06 | .5622 | 6 | .50 | .3125 | 35 | .15 | .3750 | 59 | .07 | .6004 |
|  | ***ESR2*** | | | | | | | | | | | | | | | | | | | | | | | | | | | | | | | | |
| Concentration [10^6^ per ml] | 92 | -.03 | .7662 | 30 | .10 | .6123 | 62 | .04 | .7552 | 75 | -.04 | .7341 | 17 | -.15 | .5757 | 70 | .15 | .2276 | 22 | -.09 | .7038 | 87 | .02 | .8682 | 5 | .20 | .7471 | 35 | .25 | .1530 | 57 | .00 | .9826 |
| Progressive motility [PR, %] | 92 | -.29 | **.0046** | 30 | .14 | .4512 | 62 | -.36 | **.0038** | 75 | -.24 | .0367 | 17 | -.52 | **.0310** | 70 | -.04 | .7354 | 22 | .04 | .8550 | 87 | -.25 | .0179 | 5 | .50 | .3910 | 35 | .22 | .1987 | 57 | -.44 | **.0006** |
| Total motility [PR+NP %] | 92 | -.06 | .5841 | 30 | .22 | .2375 | 62 | -.11 | .4151 | 75 | -.04 | .7119 | 17 | -.15 | .5726 | 70 | .17 | .1623 | 22 | .24 | .2780 | 87 | .01 | .9134 | 5 | .10 | .8729 | 35 | .29 | .0936 | 57 | -.18 | .1757 |
| Immotile spermatozoa [IM, %] | 92 | .05 | .6081 | 30 | -.24 | .1928 | 62 | .11 | .4151 | 75 | .04 | .7440 | 17 | .15 | .5726 | 70 | -.18 | .1401 | 22 | -.24 | .2780 | 87 | -.02 | .8812 | 5 | -.10 | .8729 | 35 | -.30 | .0754 | 57 | .18 | .1757 |
| Vitality [%] | 92 | -.09 | .4146 | 30 | .10 | .6099 | 62 | -.12 | .3636 | 75 | -.03 | .7812 | 17 | -.21 | .4276 | 70 | .18 | .1425 | 22 | -.03 | .8858 | 87 | -.02 | .8846 | 5 | .79 | .1114 | 35 | .19 | .2723 | 57 | -.15 | .2679 |
| Morphology [%] | 92 | -.33 | **.0012** | 30 | -.25 | .1825 | 62 | -.35 | **.0050** | 75 | -.31 | .0067 | 17 | -.49 | .**0463** | 70 | -.20 | .0985 | 22 | -.31 | .1624 | 87 | -.31 | .0037 | 5 | .35 | .5594 | 35 | -.14 | .4274 | 57 | -.25 | .0569 |
| Round cells [10^6^ per ml] | 92 | -.14 | .1995 | 30 | -.26 | .1665 | 62 | -.06 | .6653 | 75 | -.09 | .4317 | 17 | -.35 | .1671 | 70 | .02 | .8521 | 22 | -.22 | .3207 | 87 | -.08 | .4470 | 5 | -.58 | .3056 | 35 | -.21 | .2179 | 57 | -.04 | .7656 |
| Round cells [%] | 92 | -.18 | .0811 | 30 | -.33 | .0763 | 62 | -.18 | .1545 | 75 | -.12 | .3103 | 17 | -.45 | .0712 | 70 | -.18 | .1467 | 22 | -.37 | .0932 | 87 | -.19 | .0818 | 5 | -.67 | .2189 | 35 | -.41 | .0152 | 57 | -.13 | .3394 |
|  | ***SRC*** | | | | | | | | | | | | | | | | | | | | | | | | | | | | | | | | |
| Concentration [10^6^ per ml] | 92 | -.15 | .1429 | 29 | -.09 | .6503 | 63 | -.15 | .2558 | 74 | -.14 | .2398 | 18 | .19 | .4550 | 70 | -.19 | .1124 | 22 | .01 | .9482 | 86 | -.20 | .0690 | 6 | -.72 | .1032 | 34 | -.04 | .8190 | 58 | -.17 | .1984 |
| Progressive motility [PR, %] | 92 | -.06 | .5436 | 29 | .19 | .3246 | 63 | -.14 | .2765 | 74 | -.04 | .7283 | 18 | -.08 | .7414 | 70 | .05 | .6652 | 22 | -.25 | .2560 | 86 | -.12 | .2739 | 6 | -.17 | .7417 | 34 | .21 | .2234 | 58 | -.16 | .2346 |
| Total motility [PR+NP %] | 92 | .08 | .4695 | 29 | .03 | .8658 | 63 | .15 | .2410 | 74 | .09 | .4414 | 18 | .14 | .5752 | 70 | .12 | .3172 | 22 | .45 | **.0360** | 86 | .02 | .8761 | 6 | .58 | .2278 | 34 | .08 | .6376 | 58 | .12 | .3840 |
| Immotile spermatozoa [IM, %] | 92 | -.08 | .4320 | 29 | -.06 | .7582 | 63 | -.15 | .2410 | 74 | -.10 | .4020 | 18 | -.14 | .5752 | 70 | -.13 | .2725 | 22 | -.45 | **.0360** | 86 | -.02 | .8208 | 6 | -.58 | .2278 | 34 | -.10 | .5607 | 58 | -.12 | .3840 |
| Vitality [%] | 92 | .15 | .1456 | 29 | .15 | .4231 | 63 | .22 | .0893 | 74 | .22 | .0596 | 18 | .11 | .6683 | 70 | .14 | .2605 | 22 | .47 | **.0291** | 86 | .11 | .3276 | 6 | -.54 | .2715 | 34 | .26 | .1341 | 58 | .19 | .1520 |
| Morphology [%] | 92 | -.13 | .2182 | 29 | -.14 | .4777 | 63 | -.10 | .4556 | 74 | -.07 | .5534 | 18 | -.26 | .3057 | 70 | -.12 | .3106 | 22 | -.01 | .9730 | 86 | -.20 | .0709 | 6 | .60 | .2078 | 34 | -.07 | .6821 | 58 | -.03 | .8420 |
| Round cells [10^6^ per ml] | 92 | .04 | .7178 | 29 | .21 | .2802 | 63 | .00 | .9741 | 74 | .08 | .5251 | 18 | .15 | .5492 | 70 | .06 | .6125 | 22 | .05 | .8408 | 86 | .02 | .8513 | 6 | -.48 | .3380 | 34 | .29 | .0906 | 58 | -.03 | .8267 |
| Round cells [%] | 92 | .14 | .1961 | 29 | .20 | .3082 | 63 | .08 | .5231 | 74 | .15 | .2148 | 18 | .01 | .9740 | 70 | .19 | .1140 | 22 | -.09 | .6921 | 86 | .15 | .1719 | 6 | .18 | .7380 | 34 | .23 | .1960 | 58 | .08 | .5572 |
|  | ***PELP1*** | | | | | | | | | | | | | | | | | | | | | | | | | | | | | | | | |
| Concentration [10^6^ per ml] | 90 | .04 | .7365 | 27 | .25 | .2016 | 63 | -.09 | .4732 | 73 | .12 | .3055 | 17 | -.20 | .4389 | 68 | .12 | .3101 | 22 | -.18 | .4193 | 85 | .04 | .7426 | 5 | -.10 | .8729 | 32 | .22 | .2316 | 58 | -.10 | .4773 |
| Progressive motility [PR, %] | 90 | .07 | .4883 | 27 | .18 | .3556 | 63 | -.01 | .9506 | 73 | .10 | .3922 | 17 | .12 | .6417 | 68 | .14 | .2586 | 22 | -.03 | .8971 | 85 | .08 | .4669 | 5 | -.30 | .6238 | 32 | .13 | .4750 | 58 | .04 | .7930 |
| Total motility [PR+NP %] | 90 | -.01 | .9184 | 27 | .03 | .8880 | 63 | -.08 | .5229 | 73 | .00 | .9905 | 17 | .01 | .9776 | 68 | -.05 | .7125 | 22 | .07 | .7509 | 85 | -.02 | .8746 | 5 | -.30 | .6238 | 32 | .04 | .8100 | 58 | -.05 | .7223 |
| Immotile spermatozoa [IM, %] | 90 | .00 | .9721 | 27 | -.06 | .7814 | 63 | .08 | .5229 | 73 | -.01 | .9382 | 17 | -.01 | .9776 | 68 | .04 | .7720 | 22 | -.07 | .7509 | 85 | .01 | .9322 | 5 | .30 | .6238 | 32 | -.06 | .7351 | 58 | .05 | .7223 |
| Vitality [%] | 90 | .05 | .6371 | 27 | .01 | .9493 | 63 | .02 | .8795 | 73 | .11 | .3417 | 17 | -.04 | .8809 | 68 | -.03 | .8136 | 22 | .20 | .3628 | 85 | .05 | .6627 | 5 | -.79 | .1114 | 32 | .13 | .4748 | 58 | -.01 | .9630 |
| Morphology [%] | 90 | .09 | .3915 | 27 | -.10 | .6079 | 63 | .06 | .6580 | 73 | .13 | .2648 | 17 | .08 | .7687 | 68 | .12 | .3133 | 22 | -.14 | .5454 | 85 | .10 | .3566 | 5 | -.71 | .1817 | 32 | -.09 | .6134 | 58 | .02 | .8830 |
| Round cells [10^6^ per ml] | 90 | .07 | .5282 | 27 | .02 | .9105 | 63 | .00 | .9928 | 73 | .05 | .6691 | 17 | .21 | .4170 | 68 | .02 | .8855 | 22 | .20 | .3696 | 85 | .06 | .5800 | 5 | .37 | .5411 | 32 | .13 | .4892 | 58 | -.04 | .7820 |
| Round cells [%] | 90 | .09 | .3989 | 27 | -.17 | .3981 | 63 | .14 | .2618 | 73 | .01 | .9014 | 17 | .35 | .1691 | 68 | -.01 | .9326 | 22 | .49 | **.0211** | 85 | .08 | .4420 | 5 | .41 | .4925 | 32 | -.08 | .6742 | 58 | .11 | .3946 |

N – number of cases; R – Spearman’s rank correlation coefficient; *p*-values < 0.05 are indicated in bold.

**Supplementary Table S7.** Spearman rank order correlations of the analyzed gene ratios in subgroups distinguished according to the WHO 2010 value of particular semen parameter analyzed separately.

|  | Concentration  WHO reference [15x10^6^ per ml] | | | | | | Progressive motility  WHO reference [32%] | | | | | | Vitality  WHO reference [4%] | | | | | | Morphology  WHO reference [4%] | | | | | |
| --- | --- | --- | --- | --- | --- | --- | --- | --- | --- | --- | --- | --- | --- | --- | --- | --- | --- | --- | --- | --- | --- | --- | --- | --- |
|  | Normal | | | Abnormal | | | Normal | | | Abnormal | | | Normal | | | Abnormal | | | Normal | | | Abnormal | | |
|  | N | R | *P*-value | N | R | *P*-value | N | R | *P*-value | N | R | *P*-value | N | R | *P*-value | N | R | *P*-value | N | R | *P*-value | N | R | *P*-value |
| *ESR1* & *ESR2* | 75 | -.57 | <.**0001** | 17 | -.54 | **.0257** | 70 | -.56 | <.**0001** | 22 | -.54 | .**0091** | 87 | -.59 | <.**0001** | 5 | .05 | .9347 | 35 | -.57 | .**0003** | 57 | -.52 | <.**0001** |
| *ESR1* & *SRC* | 74 | -.16 | .1757 | 17 | -.60 | **.0117** | 69 | -.19 | .1136 | 22 | -.33 | .1300 | 85 | -.29 | .**0077** | 6 | .43 | .3991 | 34 | -.15 | .3917 | 57 | -.23 | .0822 |
| *ESR1* & *PELP1* | 73 | .13 | .2730 | 17 | .03 | .9177 | 68 | .15 | .2309 | 22 | -.12 | .5986 | 85 | .13 | .2390 | 5 | -.41 | .4925 | 32 | -.04 | .8193 | 58 | .13 | .3425 |
| *ESR2* & *SRC* | 72 | .09 | .4613 | 17 | .38 | .1380 | 68 | .07 | .5548 | 21 | .17 | .4522 | 84 | .19 | .0785 | 5 | -.30 | .6238 | 34 | -.05 | .7724 | 55 | .15 | .2645 |
| *ESR2* & *PELP1* | 72 | -.28 | **.0158** | 17 | -.43 | .0869 | 67 | -.29 | .**0167** | 22 | -.49 | .**0199** | 84 | -.30 | .**0048** | 5 | -.90 | .**0374** | 32 | -.22 | .2223 | 57 | -.33 | .**0126** |
| *SRC* & *PELP1* | 71 | .30 | **.0108** | 17 | .06 | .8148 | 67 | .32 | .**0093** | 21 | .16 | .5022 | 83 | .26 | .**0185** | 5 | .10 | .8729 | 32 | .50 | .**0034** | 56 | .19 | .1708 |

N – number of cases; R – Spearman’s rank correlation coefficient; *p*-values < 0.05 are indicated in bold.

| **MIQE checklist and data set** | | | |
| --- | --- | --- | --- |
| **ITEM TO CHECK** | **IMPORTANCE** | **Comments** | |
| **EXPERIMENTAL DESIGN** |  |  | |
| Definition of experimental and control groups | **E** | Provided throughout manuscript | |
| Number within each group | **E** | Provided throughout manuscript | |
| Assay carried out by core lab or investigator's lab? | D | Yes | |
| Acknowledgement of authors' contributions | D | Yes | |
| **SAMPLE** |  |  | |
| Description | **E** | Materials and methods | |
| Volume/mass of sample processed | D | Materials and methods | |
| Microdissection or macrodissection | **E** | NA | |
| Processing procedure | **E** | Materials and methods | |
| If frozen - how and how quickly? | **E** | Materials and methods | |
| If fixed - with what, how quickly? | **E** | NA | |
| Sample storage conditions and duration (especially for FFPE samples) | **E** | Materials and methods | |
| **NUCLEIC ACID EXTRACTION** |  |  | |
| Procedure and/or instrumentation | **E** | Materials and methods | |
| Name of kit and details of any modifications | **E** | Materials and methods | |
| Source of additional reagents used | D | Materials and methods | |
| Details of DNase or RNAse treatment | **E** | Materials and methods | |
| Contamination assessment (DNA or RNA) | **E** | Materials and methods | |
| Nucleic acid quantification | **E** | Materials and methods | |
| Instrument and method | **E** | Materials and methods | |
| Purity (A260/A280) | D | Materials and methods | |
| Yield | D | Results and Supplemental Tables | |
| RNA integrity method/instrument | **E** | Materials and methods | |
| RIN/RQI or Cq of 3' and 5' transcripts | **E** | NA | |
| Electrophoresis traces | D | Materials and methods | |
| Inhibition testing (Cq dilutions, spike or other) | **E** | NA | |
| **REVERSE TRANSCRIPTION** |  |  | |
| Complete reaction conditions | **E** | Materials and methods | |
| Amount of RNA and reaction volume | **E** | Materials and methods | |
| Priming oligonucleotide (if using GSP) and concentration | **E** | NA | |
| Reverse transcriptase and concentration | **E** | Materials and methods | |
| Temperature and time | **E** | Materials and methods | |
| Manufacturer of reagents and catalogue numbers | D | Materials and methods | |
| Cqs with and without RT | D* | Throughout entire manuscript | |
| Storage conditions of cDNA | D | -20^°^C (not longer than one week) or immediate use | |
| **qPCR TARGET INFORMATION** |  |  | |
| If multiplex, efficiency and LOD of each assay. | **E** | NA | |
| Sequence accession number | **E** | NA | |
| Location of amplicon | D | NA | |
| Amplicon length | **E** | Materials and methods | |
| In silico specificity screen (BLAST, etc) | **E** | Materials and methods | |
| Pseudogenes, retropseudogenes or other homologs? | D | NA | |
| Sequence alignment | D | Materials and methods | |
| Secondary structure analysis of amplicon | D | NA | |
| Location of each primer by exon or intron (if applicable) | **E** | Materials and methods | |
| What splice variants are targeted? | **E** | Materials and methods | |
| **qPCR OLIGONUCLEOTIDES** |  |  | |
| Primer sequences | **E** | Materials and methods or NA | |
| RTPrimerDB Identification Number | D | NA | |
| Probe sequences | D | Commercial pre-designed assays | |
| Location and identity of any modifications | **E** | Materials and methods | |
| Manufacturer of oligonucleotides | D | Materials and methods | |
| Purification method | D | HPLC | |
| **qPCR PROTOCOL** |  |  | |
| Complete reaction conditions | **E** | Materials and methods | |
| Reaction volume and amount of cDNA/DNA | **E** | Materials and methods | |
| Primer, (probe), Mg++ and dNTP concentrations | **E** | Materials and methods | |
| Polymerase identity and concentration | **E** | Materials and methods | |
| Buffer/kit identity and manufacturer | **E** | Materials and methods | |
| Exact chemical constitution of the buffer | D | NA | |
| Additives (SYBR Green I, DMSO, etc.) | **E** | NA | |
| Manufacturer of plates/tubes and catalog number | D | Materials and methods | |
| Complete thermocycling parameters | **E** | Materials and methods | |
| Reaction setup (manual/robotic) | D | Manual | |
| Manufacturer of qPCR instrument | **E** | Materials and methods | |
| **qPCR VALIDATION** |  |  | |
| Evidence of optimisation (from gradients) | D | NA | |
| Specificity (gel, sequence, melt, or digest) | **E** | NA | |
| For SYBR Green I, Cq of the NTC | **E** | NA | |
| Standard curves with slope and y-intercept | **E** | Materials and methods | |
| PCR efficiency calculated from slope | **E** | Materials and methods/Standard curve | |
| Confidence interval for PCR efficiency or standard error | D | Materials and methods/Standard curve | |
| r2 of standard curve | **E** | Standard curve | |
| Linear dynamic range | **E** | NA | |
| Cq variation at lower limit | **E** | Materials and methods/Duplicates | |
| Confidence intervals throughout range | D | NA | |
| Evidence for limit of detection | **E** | Yes (Decimal dilutions) | |
| If multiplex, efficiency and LOD of each assay. | **E** | NA | |
| **DATA ANALYSIS** |  |  | |
| qPCR analysis program (source, version) | **E** | Materials and methods | |
| Cq method determination | **E** | Yes | |
| Outlier identification and disposition | **E** | Yes | |
| Results of NTCs | **E** | Yes | |
| Justification of number and choice of reference genes | **E** | Materials and methods | |
| Description of normalization method | **E** | Materials and methods | |
| Number and concordance of biological replicates | D | Materials and methods | |
| Number and stage (RT or qPCR) of technical replicates | **E** | Materials and methods | |
| Repeatability (intra-assay variation) | **E** | Yes. | |
| Reproducibility (inter-assay variation, %CV) | D | NA | |
| Power analysis | D | NA | |
| Statistical methods for result significance | **E** | Throughout Manuscript, Supplementary materials | |
|  |  |  | |

E – essential information, D – desirable information, NA – not applicable or not available
